# The α-Gal epitope - the cause of a global allergic disease

**DOI:** 10.3389/fimmu.2024.1335911

**Published:** 2024-01-22

**Authors:** Marija Perusko, Jeanette Grundström, Maria Eldh, Carl Hamsten, Danijela Apostolovic, Marianne van Hage

**Affiliations:** ^1^ Division of Immunology and Allergy, Department of Medicine Solna, Karolinska Institutet, and Karolinska University Hospital, Stockholm, Sweden; ^2^ Center for Molecular Medicine, Karolinska Institutet, Stockholm, Sweden; ^3^ Innovative Centre of the Faculty of Chemistry, University of Belgrade, Belgrade, Serbia

**Keywords:** α-Gal syndrome, galactose α-1,3-galactose, α-Gal epitope, carbohydrate epitope, mechanisms, ticks

## Abstract

The galactose-α-1,3-galactose (α-Gal) epitope is the cause of a global allergic disease, the α-Gal syndrome (AGS). It is a severe form of allergy to food and products of mammalian origin where IgE against the mammalian carbohydrate, α-Gal, is the cause of the allergic reactions. Allergic reactions triggered by parenterally administered α-Gal sources appear immediately, but those triggered via the oral route appear with a latency of several hours. The α-Gal epitope is highly immunogenic to humans, apes and old-world monkeys, all of which produce anti-α-Gal antibodies of the IgM, IgA and IgG subclasses. Strong evidence suggests that in susceptible individuals, class switch to IgE occurs after several tick bites. In this review, we discuss the strong immunogenic role of the α-Gal epitope and its structural resemblance to the blood type B antigen. We emphasize the broad abundance of α-Gal in different foods and pharmaceuticals and the allergenicity of various α-Gal containing molecules. We give an overview of the association of tick bites with the development of AGS and describe innate and adaptive immune response to tick saliva that possibly leads to sensitization to α-Gal. We further discuss a currently favored hypothesis explaining the mechanisms of the delayed effector phase of the allergic reaction to α-Gal. We highlight AGS from a clinical point of view. We review the different clinical manifestations of the disease and the prevalence of sensitization to α-Gal and AGS. The usefulness of various diagnostic tests is discussed. Finally, we provide different aspects of the management of AGS. With climate change and global warming, the tick density is increasing, and their geographic range is expanding. Thus, more people will be affected by AGS which requires more knowledge of the disease.

## Introduction

The α-Gal syndrome (AGS) is an increasingly recognized public health issue and the most enigmatic food allergy. It was discovered more than a decade ago and represents a severe form of allergy to mammalian meat, which has been reported worldwide (1). The syndrome results from IgE-mediated responses to galactose-α-1,3-galactose (α-Gal), a sugar moiety covalently bound to proteins and lipids of mammalian origin. Numerous foods and pharmaceuticals with components of mammalian origin contain α-Gal and may trigger reactions, which is why this allergic disease is known as the α-Gal syndrome, AGS.

AGS challenges the current paradigm of food allergy due to several features: I) It is the first known allergic disease where a carbohydrate solely is the cause of IgE-mediated allergic reactions. In conventional allergies, IgE antibodies are directed to protein epitopes, and before the discovery of AGS, IgE against carbohydrates were considered of low or no clinical importance. II) Patients with AGS develop allergic symptoms several hours (typically 2 – 6 h) after consumption of α-Gal-containing foods, which contrasts with conventional food allergies where patients react to allergenic food within minutes following ingestion. This delay is not yet understood. III) The sensitizing agents are several tick species, but not the food itself. The patients develop IgE against α-Gal after being tick bitten several times with the consequence that all α-Gal containing food and pharmaceutical sources are potentially allergenic. Tick exposure usually precedes the onset of AGS by 1 to 6 months (2). IV) AGS affects mostly middle-aged patients, although children may also develop the disease. V) Individuals expressing the B-antigen (blood group B/AB) seem to have a significantly lower risk of developing AGS due to the structural similarity between α-Gal and the B-antigen resulting in immune tolerance. The prevalence of AGS is currently unknown, but in 2023 the U.S. Centers for Disease Control and Prevention estimated that as many as 450,000 people might have been affected in the US (3). The number of suspected AGS cases has increased substantially since 2010, and geographic locations with established populations of certain tick species are most affected, although suspected AGS cases were also identified in areas outside of ticks’ range (3).

## IgE antibody responses to carbohydrates

Protein glycosylation is among the most common posttranslational modifications, and it is highly conserved among many organisms. Glycosylation contributes significantly to protein characteristics and functions (solubility, stability, adherence, targeted transport, activity, etc.). Common glycosylation patterns found in plants, invertebrates, and non-primate mammals are absent in humans, which makes them immunogenic. Many allergens, especially those from pollen, plant food, and Hymenoptera venom are N-glycosylated with carbohydrate determinants with IgE binding properties. These carbohydrates have a common structure of a core (two N-acetylglucosamine (GlcNAc) sugars with two or three terminal mannose residues) with β1–2 linked xylose and/or α1–3 linked fucose [Reviewed in (4)], and they exhibit wide cross-reactivity. Therefore, they are termed as cross-reactive carbohydrate determinants (CCDs). Sera from patients with anti-CCD IgE antibodies demonstrate high cross-reactivity between inhalant (e.g., pollen allergens) and plant food allergens (5, 6). However, the anti-CCD IgE has little or no clinical relevance, as it does not contribute to allergic symptoms (5, 6). The overall prevalence of IgE to CCDs among pollen and food allergic subjects is around 20% (7, 8). Therefore, some diagnostic multiplex IgE assays offer either a specific testing for common CCDs in parallel with protein allergens, or even include a CCD blocking step before testing for protein specific IgE. Inhibition of IgE binding against CCDs leads to a significant reduction in false-positive *in vitro* diagnostic tests (9).

The biological relevance with respect to allergenic activity of CCDs has been demonstrated in a few allergens only (10–15). However, no plant-derived CCDs have been associated with anaphylaxis (16). The discovery of AGS, in which IgE against a mammalian CCD (α-Gal) solely mediates allergic reactions including severe anaphylaxis, contrasts with traditional understanding of carbohydrate IgE responses in allergy. The clinical significance of the α-Gal epitope led to the recent incorporation of glycan epitopes into the WHO/IUIS Allergen database (4).

## The α-Gal epitope – a strong immunogen

The α-Gal epitope (which naturally occurs as Galα1–3Galβ1–4GlcNAc–R) is a common N-terminal glycosylation in all non-primate mammals and new-world monkeys (17). It is synthesized by the glycosylation enzyme α1–3galactosyltransferase (α1–3GT) (17). This enzyme links the galactose residue to the N–acetyllactosaminyl group (Galβ1–4GlcNAc–R) using uridine diphosphate galactose as the sugar donor, thus forming the trisaccharide Galα1–3Galβ1–4GlcNAc–R on various glycans (18). The α-Gal epitope is one of the most abundant carbohydrate epitopes on glycoproteins and glycolipids of non-primate mammals. In humans, apes, and old-world monkeys the gene for the α1–3GT enzyme has been inactivated due to a premature stop codon, and therefore they do not express the α-Gal epitope (17).

The α-Gal epitope is highly immunogenic for humans, apes and old-world monkeys, all of which produce natural anti-α-Gal antibodies (anti-Gal) of the IgM, IgA and IgG subclasses (19). Due to gut microbiota expressing α-Gal, anti-Gal antibodies are one of the most abundant types of antibodies in humans (20, 21), constituting 0.2 - 1.0% of total immunoglobulins (22, 23). Anti-Gal antibodies are characterized by a preferential use of Ig variable region V3 family genes in the heavy chain (24–26), where a germline encoded tryptophan in position 33 of the complementarity determining region is essential for the binding to α-Gal (26). The formation of anti-Gal antibodies begins in the first few months of life and the IgM isotype rises faster and steeper compared to IgG and IgA until 2 years of age (27). It is believed that anti-Gal antibodies function in humans as a barrier to zoonotic infections by enveloped viruses produced in hosts synthesizing α-Gal epitopes (28). Both the α-Gal epitope and the anti-Gal antibodies have been well studied and the focus of research for the last 50 years due to their role in the initiation of hyperacute rejection of xenografts (29). Human anti-Gal IgG antibodies recognize α-Gal on endothelial cells of an organ xenotransplant and initiate complement-mediated cell lysis leading to rapid rejection of xenografts. New light has been shed on the α-Gal epitope after the discovery of anti-Gal IgE antibodies and their role in allergic reactions including life-threatening anaphylaxis (1).

## The α-Gal epitope structurally resembles the blood group B antigen

**Figure 1:**
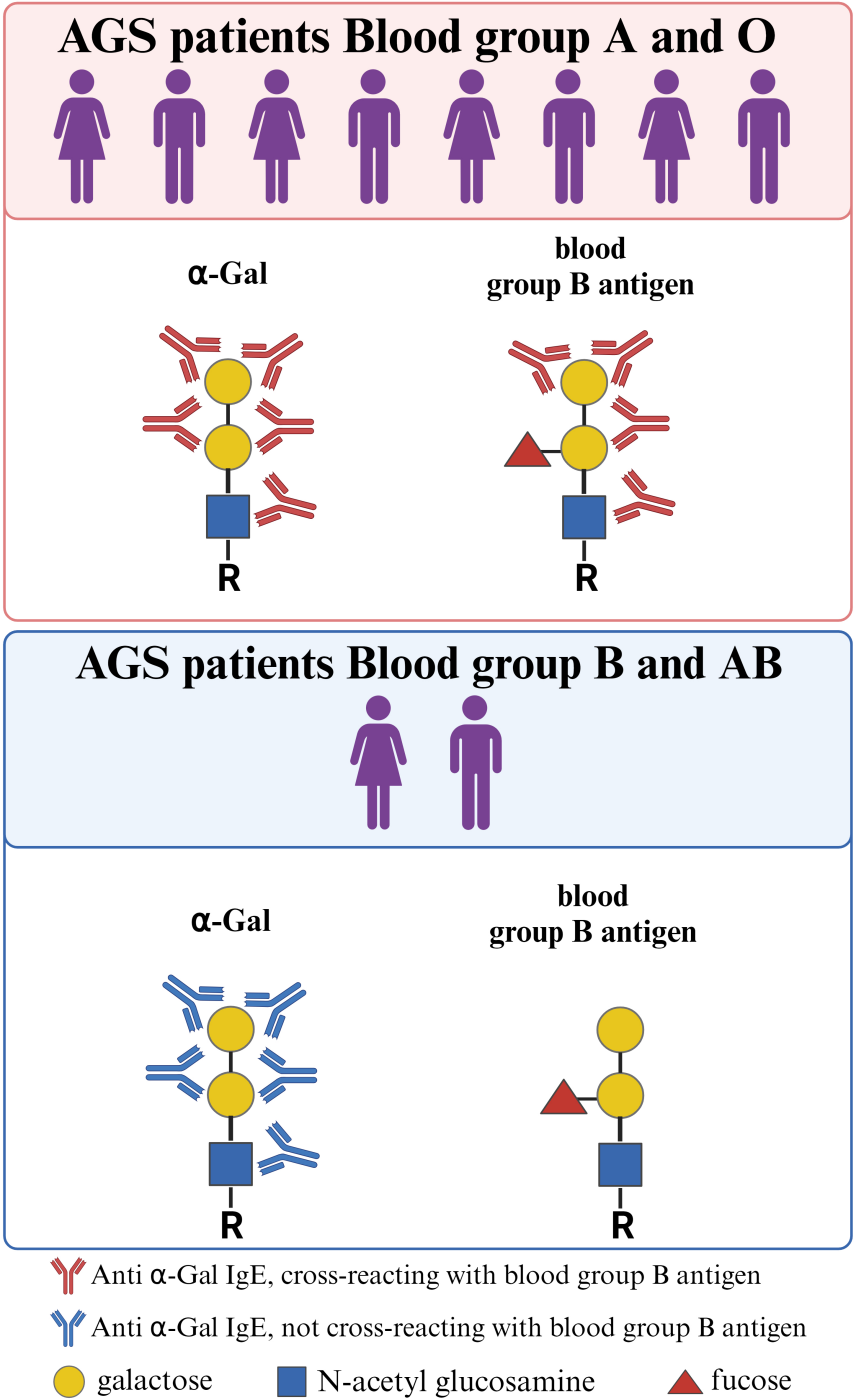
Structural resemblance between α-Gal and the blood group B antigen. Regardless of blood type, individuals may develop AGS. However, individuals with blood group B/AB are underrepresented among AGS patients, as the structural similarity between the α-Gal epitope and the blood group B antigen confers protection against developing anti-α-Gal IgE antibodies. In addition, anti-α-Gal IgE antibodies from AGS patients with blood group A or 0 often cross-react with the blood group B antigen. This cross-reactivity is absent in AGS patients with blood group B/AB. Created with BioRender.com.

The α-Gal oligosaccharide structurally resembles the blood group B antigen, where the only difference is an α1–2 linked fucosyl group on the penultimate galactosyl group in the B antigen. Indeed, anti-Gal antibodies from healthy individuals can cross-react with blood group B antigen (30). Subjects with B or AB blood groups, although they possess α-Gal-specific antibodies, lack those which cross-react with the B antigen (30). Similarly, patients with AGS or α-Gal sensitized subjects typically have IgE antibodies which cross-reacts with both α-Gal and the B antigen, except for subjects expressing the B antigen (**Figure 1**) (31, 32). The blood group B or AB also seems to be protective for developing AGS (33, 34). The molecular mechanisms underlying the protective effect of blood type B need further research. Whether broader specificities in subjects with A or O blood type act as drivers of α-Gal sensitization and AGS remains to be answered. Interestingly, although AGS patients develop IgE against α-Gal, they rarely have IgE against other mammalian carbohydrate epitopes or even CCDs (35, 36).

## The α-Gal epitope confers allergenicity to diverse molecules

Due to the broad abundance of α-Gal on glycolipids and glycoproteins in mammals, an almost infinite number of these molecules can be the cause of an allergic reaction to food and products of mammalian origin. Among the first proteins determined to contain α-Gal and bound IgE from patients with beef allergy, were the high molecular weight beef laminin γ-1 and collagen α-1 (VI) chain (37). We identified seven α-Gal containing beef proteins that bound AGS patient IgE, whereof four (creatine kinase M type, aspartate aminotransferase, α- and β- enolase) were stable after heat treatment (38). Furthermore, bovine gamma globulin (BGG, Bos d 7) was determined to be the most recognized α-Gal containing beef protein among AGS patients (39), a protein which is also the cause of beef allergy. BGG is also present in bovine milk. Perusko et al. have shown that BGG together with the milk proteins lactoferrin and lactoperoxidase, have allergenic activity in AGS patients (40). Interestingly, sheep immunoglobulins seem to have more α-Gal than bovine (41), which is demonstrated in a case study where two AGS patients reacted to cheese made from sheep’s milk but not cow’s milk (42). Pork kidney has been recognized as the meat source containing the highest amount of α-Gal and the most potent trigger of AGS symptoms (43). This is reflected in immunohistochemical staining of α-Gal in pork and beef kidney and muscle, where pork kidney stains the strongest while α-Gal was barely detected in pork muscle (44). Two pork kidney proteins with allergenic activity, aminopeptidase N and angiotensin-converting enzyme 1, have been identified and the allergenic activity was shown to be due to α-Gal (45). Interestingly, gelatin obtained from bovine or porcine sources contains small amounts of α-Gal (46). Consumption of gelatin-containing candy after exercise has been shown to induce a delayed allergic reaction in one AGS patient (47), emphasizing that all forms of food from mammalian sources need to be considered for avoidance in AGS patients.

The α-Gal epitope is also present in numerous pharmaceuticals with some mammalian content. Indeed, AGS was discovered when a monoclonal antibody, cetuximab, used in colorectal and head and neck cancer treatment induced anaphylaxis in 22% of patients in certain geographic locations (48). Cetuximab was produced in a mouse cell line and investigations showed that the target of the IgE binding was an oligosaccharide, α-Gal, located on the asparagine at position 88 in the Fab region of the heavy chain (49). Similarly, antivenoms, i.e. mammalian anti-venom Fab are also a possible risk for AGS patients. Antivenoms contain α-Gal and can activate basophils *in vitro* (50). The antivenom FabAV has been involved in immediate hypersensitivity reactions in several patients (51, 52), and the α-Gal on the Fab can activate basophils *in vitro* (52). Gelatin of porcine or bovine origin is present in some vaccine formulations (MMR, Zoster, varicella vaccine) and AGS patients’ IgE binding to these vaccines has been demonstrated (53, 54). These vaccines possess capacity to activate AGS patients’ basophils (55). Furthermore, there are case reports on anaphylaxis upon parenteral administration of zoster vaccine, or a combination of MMR and varicella vaccine in adult and pediatric AGS patients (53, 54). Gelatin-based colloid plasma substitute (Gelofusine) may also induce anaphylaxis in AGS patients (56).

**Figure 2:**
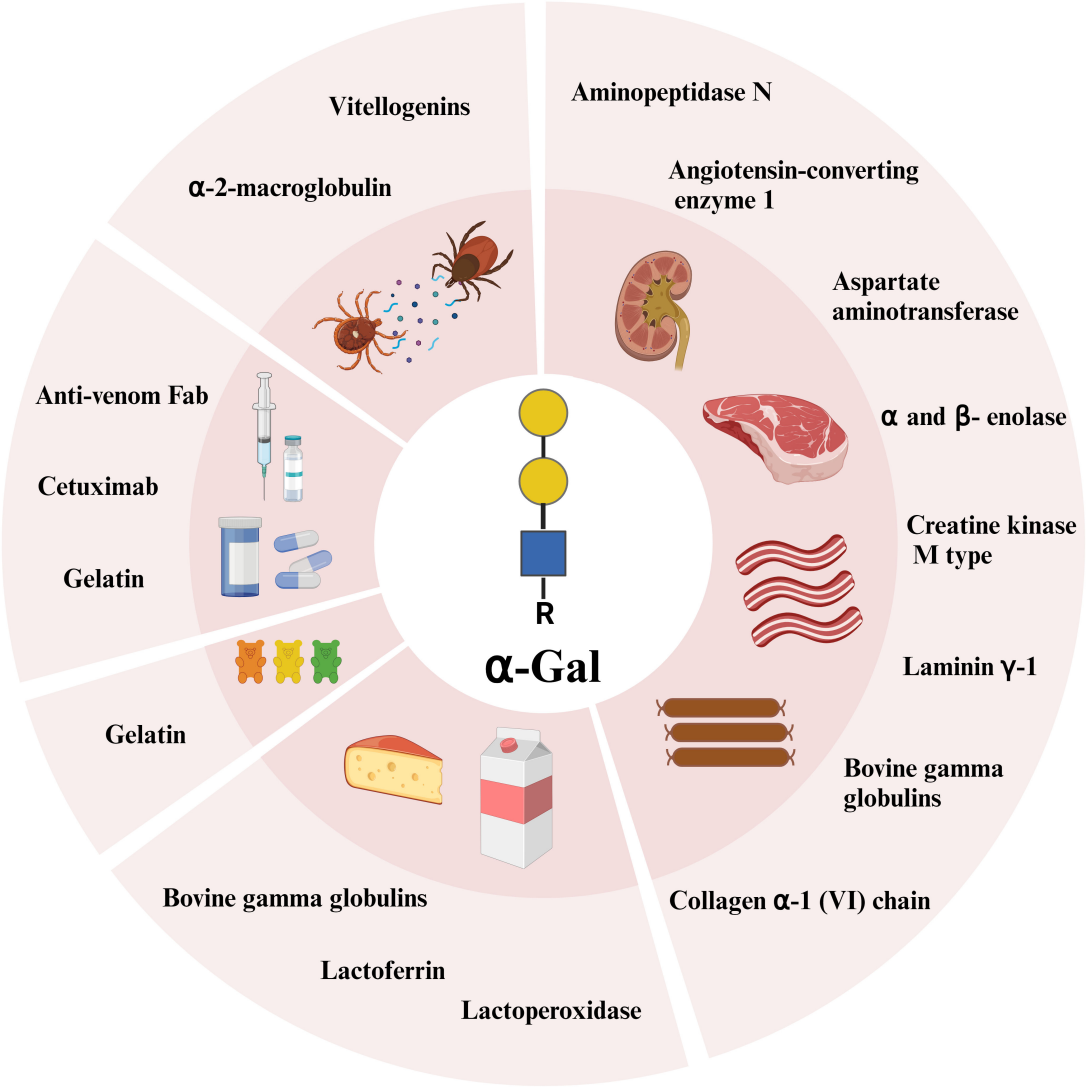
α-Gal carrying glycoproteins identified so far from innards, beef, milk, sweets, pharmaceuticals, and ticks. Created with BioRender.com.

Proteins containing α-Gal are also present in ticks. In extract from the European tick *Ixodes ricinus*, two major groups of proteins were found to carry α-Gal, vitellogenins and α-2-macroglobulin (57). Many proteins and enzymes involved in carbohydrate metabolism were found as α-Gal carrying candidates in the American tick species *Amblyomma americanum* and *Ixodes scapularis* (58). Although the extract from *I. ricinus* as well as saliva from *A. americanum* and *I. scapularis* were shown to activate AGS patients’ basophils (57, 58), the allergenic activity of individual α-Gal-carrying proteins has not been assessed. **Figure 2** summarizes discussed α-Gal-carrying molecules.

## Association of AGS with tick bites

The association of AGS with tick bites has been postulated since the first reports of AGS, which was based on the geographical overlap with tick endemic areas in the southeastern US and coastal eastern Australia (1, 2, 59). Also, documented AGS cases from Australia were characterized by large local reactions to tick bites (2). Accumulated evidence over the past 15 years has strengthened the hypothesis that repeated tick bites lead to the development of AGS in susceptible individuals, although the exact mechanisms are not well understood. Several tick species such as *I. holocyclus* in Australia, *A. americanum* in North America, *I. ricinus* in Europe, *Haemaphysalis longicornis* and *Amblyomma testudinarium* in Asia and *Amblyomma sculptum* in South America have been linked with the development of AGS (33, 59–63). The first potential explanation for the relationship between tick exposure and sensitization to α-Gal came from the early work of Hamsten et al., who demonstrated the presence of α-Gal within the gastrointestinal tract of *I. ricinus* ticks (64). This opened the question of the origin of α-Gal within the ticks: biosynthesis by ticks themselves, transfer from a mammalian host organism during feeding, or biosynthesis by midgut microbiota. The presence of α-Gal has been demonstrated in *A. americanum* tick saliva and salivary glands when fed on human blood which lack α-Gal (58). The α-Gal moieties in tick salivary glands were colocalized to the salivary secretory vesicles of the salivary acini, confirming the secretory nature of α-Gal-containing antigens in ticks (58). So far, the enzyme α1–3GT has not been identified in ticks. However, three other galactosyltransferase encoding genes have been identified in the *I. scapularis* genome that are possibly involved in the α-Gal synthesis pathway (65). *I. scapularis* has not been linked to AGS (66), and similar genes in AGS related tick species are still to be found. An immunoproteomics-based study by Apostolovic et al. revealed the presence of α-Gal-carrying proteins in both larvae and adult *I. ricinus* ticks (57). Fisher and coworkers performed an immunohistochemistry study showing that α-Gal was present in fed and unfed *I. ricinus* ticks. Furthermore, they presented evidence that the metabolic incorporation of constituents of a mammalian blood meal as well as the endogenous production contribute to the presence of α-Gal epitopes in tick tissue and saliva (67). Findings of intact host proteins (e.g. host immunoglobulins) adsorbed in the tick midgut and their later secretion into the tick saliva support the hypothesis that at least some α-Gal present in tick saliva originates from a mammalian blood meal (68). The role of tick microbiome for the presence of α-Gal in tick saliva, although suggested, is less clear (66). No published reports have provided evidence that microbes common to relevant ticks express α-Gal, nor contribute to α-Gal sensitization (66, 69–71). So far, it has been shown that the tick bacterium *Anaplasma phagocytophilum* has the capacity to increase the content of α-Gal in infected tick cells (65). However, the individual significance of the α-Gal epitope in tick saliva originating from different sources for the development of AGS has not been clarified yet.

The observed relation between tick bites and AGS has raised the question whether other biting or stinging arthropods may be associated with AGS as well. It is currently suspected that Trombiculidae larvae, known as chiggers, who bite and parasitically feed on mammals, may also be implicated in α-Gal sensitization and development of AGS (72). A study by Choudhary et al. demonstrated that AGS patients from the US were 5 times more likely to be sensitized to hymenoptera venom compared to controls (73). Venom allergy has also been reported among meat allergic patients from Turkey (74). More than 50% of our AGS patients were sensitized to wasp, however the IgE levels to wasp were low. We noted that wasp sensitization mainly occurred among AGS patients with higher IgE levels to α‐Gal and was probably due to cross-reactivity between wasp and tick proteins (75). Positive associations between sensitization to α-Gal and bee and wasp venom were also found in an AGS cohort from the US (34).

## Mechanisms of sensitization to α-Gal

Tick bites are thought to contribute directly to IgE class switching in AGS, and tick saliva is essential in this process. Saliva from several different tick species has been shown to have immunosuppressive effects (76–81), and the high amount of prostaglandin E2 in tick saliva has been indicated as a possible mediator of these effects (82). The early host response to tick bite is dominated by innate immune cells while lymphocytes increase after more than 24 h of tick attachment (83). The infiltration of immune cells is also more pronounced in skin from humans that have been previously tick bitten (63, 84), and especially infiltration of basophils is stronger in subjects that have been tick bitten more than one time (63, 85). In addition, CD4 T cells present at the bite site have an increased Th2/Th1 cytokine expression ratio in subjects that have been tick bitten several times, and the IgE level to α-Gal increases with the number of tick bites (63, 85).

*I. ricinus* salivary gland extract inhibits T cell proliferation and polarizes the cytokine profile towards Th2 in PBMC culture (86). The ability of tick saliva and salivary gland extract to skew immune responses towards Th2 type of cytokines (76–79, 86) suggests that T cells can be involved in the induction of AGS. The α1-3GT knockout mouse model (87) has been used for studying the immune response to α-Gal. These mice spontaneously develop anti-Gal antibodies, similar to humans (87). T cells from α1-3GT KO mice are reactive to α-Gal containing xenopeptides as a result of antigen presentation by α-Gal recognizing B cells, but removal of α-Gal does not affect this response (88). However, T cell help was essential for development of the anti-Gal response (88, 89). Similarly, in humans, CD4+ T cells proliferate more to wild type pig PBMCs than to the α1-3GT KO counterpart (90). We have recently reported that T cells from AGS patients proliferate in response to tick extract and secrete the Th2 cytokines IL-4, IL-13, IL-3 and IL-31, but that this is not dependent on α-Gal (91). At the same time, B cell proliferation to tick extract was α-Gal dependent and required T cell help (91).

Since all immunocompetent humans produce anti-Gal in response to the gut microbiota, α-Gal-specific memory B cells (MBC) of the IgM and IgG isotypes exist before induction of AGS. In humans, the presence of IgE+ MBCs is unclear. However, allergen-specific IgG+ MBC have been clonally linked to IgE+ plasma blasts (92), and IgG+ MBC that differentiate into IgE+ plasma cells have been shown to have a specific surface phenotype, expressing CD23 and the IL-4R (93–95). In AGS patients, no α-Gal-specific IgE+ B cells were found in circulation, but α-Gal-specific B cells had similar usage of B cell receptor heavy chain V genes as healthy individuals, that were mainly of the IgM isotype (26). However, there is evidence that human IgE+ B cells mainly seem to have differentiated from IgG1+ B cells through sequential class-switching (92, 96, 97). In AGS patients, anti-Gal is more often of the IgG1 isotype than in healthy individuals (31, 32, 39, 98). Whether this is due to an already developed allergenic response towards α-Gal or if these individuals already have a higher level of anti-Gal IgG1 before sensitization is unknown. The anti-Gal IgE is bound by FcϵRI on circulating basophils and tissue resident mast cells, which will exert their effector functions and initiate an allergic reaction when α-Gal is encountered.

## Mechanisms of the effector phase allergic reaction to α-Gal

The IgE-mediated immediate-type allergic reaction with a delayed onset of 2–6 h following ingestion of mammalian meat is a feature exclusive to AGS. Several lines of evidence show that IgE to α-Gal is indeed causal for allergic reactions in AGS. *In vitro* stimulation of patients’ basophils with α-Gal-carrying proteins or lipids leads to basophil activation within 30 minutes, which corresponds to kinetics of other immediate-type food allergies (40, 45, 99, 100). Open food challenges performed with beef or pork meat in 12 AGS patients demonstrated that *in vivo* basophil activation occurs in the same time frame as appearance of clinical symptoms (101). Furthermore, *in vivo* reactions during intradermal skin test occur rapidly (102). Finally, if patients are subjected to intravenous injection of α-Gal, the allergic reaction is immediate (49). Taken together, it seems that the latency in symptom appearance reflects the time it takes for the allergen to reach the circulation. Indeed, Eller et al. showed that α-Gal appears in serum of healthy individuals several hours after ingesting pork kidney, reflecting the clinical situation of AGS (several hours of latency) (103). Different explanations for the time delay have been suggested but the field is far from fully investigated. One hypothesis is that the allergic reactions are mediated by α-Gal-carrying glycolipids (16, 102). Lipids are metabolized more slowly than proteins. Ingested glycolipids are first digested and then absorbed by the intestinal epithelial cells and packed into chylomicrons. Chylomicrons are too large to enter the circulation directly, but they can pass through fenestrations in the lacteals, which bring them into the lymphatic system. From the lymphatic system, chylomicrons enter the blood circulation at the left subclavian vein. In the bloodstream, exchange of proteins and lipids occurs between chylomicrons and smaller lipoprotein particles such as high-density lipoproteins (HDL). HDL carrying ingested glycolipids can penetrate to the tissues where resident mast cells loaded with anti-Gal IgE can be activated. Thus, transport of α-Gal-carrying glycolipids from the ingestion site to the effector cells at distant sites would take 4 – 5 hours (104), explaining the delay in symptom appearance. Metabolomic profiling of AGS patients and control subjects before and after oral pork challenge revealed alterations in lipid and fatty acid metabolism that are consistent with the clinical delay (105). Also, in support of the glycolipid hypothesis are the results of a recent study which used Caco-2 cell line as a model system of intestinal barrier (106). The authors performed *in vitro* digestion of beef lipid and protein extracts and stimulated Caco-2 cells with the digestion products. They found that only α-Gal bound to lipids, but not to proteins, passed the model system of intestinal barrier and activated basophils of an AGS patient (106). Furthermore, the α-Gal epitope on the natural glycolipid isoglobotrihexosylceramide (iGb3) was recognized by AGS patients’ IgE, and the synthetic homolog of iGb3, PBS-113, was a potent activator of *in vitro* sensitized basophils (100). In addition, it was shown that anti-Gal IgE from AGS patients bound to α-Gal-carrying glycolipid complexed with human CD1d, and thus antigen presentation of dietary lipid through CD1 molecules may represent a mechanism of delayed food allergy (107).

Although the glycolipid hypothesis sounds convincing, it cannot explain for example, delayed (up to 11h) anaphylactic reactions after ingestion of gelatin-containing candies which do not contain α-Gal-carrying lipids, but only α-Gal-carrying protein (47, 108). Thus, it is likely that both α-Gal-carrying lipids and proteins contribute to allergic manifestations. Recent literature data has shown that α-Gal is more abundantly expressed on glycoproteins than on glycolipids from pork kidney and beef (109). Furthermore, protein extracts from both pork kidney and beef had higher *in vitro* and *ex vivo* allergenicity than lipid extracts. The authors suggested a major role of glycoproteins in delayed anaphylaxis upon consumption of these food sources (109). In addition, it was shown that α-Gal glycosylation hampered transcytosis of the protein through the Caco-2 monolayer (110).

Other immunological mechanisms that include non-IgE pathways could also play a role in AGS. It is well recognized that anti-Gal IgG increases in parallel with anti-Gal IgE (31, 32, 39), and the alternative pathway of allergic response and/or anaphylaxis mediated by IgG and its Fc gamma receptor could take place (111, 112). Furthermore, anti-Gal IgG could form immune complexes with α-Gal carrying proteins and/or lipids which would precipitate in various tissues such as skin or joints and trigger the classical complement pathway. Complement activation may further induce mast cell degranulation (113). Indeed, this mechanism could explain why some AGS patients suffer joint pain and arthritis (114–116).

## Clinical manifestations

In most cases, AGS develops in middle-aged patients who have previously tolerated mammalian food. A systematic review of the literature has shown that the mean age of AGS cases was 51.3 (SD = 16.7, range 5–85, n = 229) (117). Similarly, increased sensitization to α-Gal was noted more frequently in subjects aged over 50 years (71). However, AGS has also been reported among pediatric patients (118, 119). It remains an open question if pediatric cases are underreported or if qualitative and/or quantitative differences in Th2 type responses between the young and aging immune system contribute to a higher predisposition of developing AGS in adulthood. Indeed, beginning with the sixth decade of life, the human immune system undergoes aging-related changes (120, 121).

The AGS symptoms may vary from urticaria, angioedema, gastrointestinal (GI) symptoms with abdominal pain, vomiting, nausea, and diarrhea to anaphylaxis. In our Swedish cohort of AGS patients, as many as 90% of patients suffered from urticaria, 74% from GI symptoms, 60% from angioedema and 47% from anaphylaxis (122). A similar study in the US reported urticaria in 93% of cases, GI symptoms in 64% and anaphylaxis in 60% (34). Patients often experience a combination of symptoms, and in the Swedish cohort the most common combination of symptoms included anaphylaxis, angioedema, urticaria and GI symptoms (122). GI symptoms are a common manifestation of food allergies. However, isolated GI symptoms without involvement of skin or cardiopulmonary symptoms are rather uncommon for food allergies. The AGS phenotype with GI symptoms only has been described in 21% of AGS cases in a black African cohort of 131 patients (119). However, it seems that the GI only phenotype is more common among black African children (median age 12 years) than in older white Americans (median age 37 years) (123). Similarly, a case report on three AGS pediatric patients described their phenotypes as non-anaphylactic GI only or GI predominant (124). Furthermore, the GI only phenotype manifests with a shorter delay (median 90 min) in comparison to systemic symptoms (median 120 min) (123). A small group of Swedish patients (8/128) reported only GI symptoms, and their α-Gal IgE levels and total IgE levels were similar to the other patients (122).

Since many AGS patients suffer from urticaria, distinguishing AGS from chronic spontaneous urticaria (CSU) can be challenging. As many as 31% (9 out of 29) of patients with CSU presenting at the University of Virginia Allergy Clinic were found to have IgE to α-Gal and experienced a complete remission of their symptoms after avoidance of mammalian meat or mammalian-derived products (125). α-Gal sensitization was observed only in a small fraction of Danish CSU patients (126). However, in a cohort of German patients from the Berlin area, with moderate-to-severe CSU, we found that an allergic response to α-Gal is highly unlikely to be an unrecognized cause of CSU (127). About 50% of patients report manifestations of life-threatening anaphylaxis (34, 122). Pattanaik et al. have, in a retrospective analysis of anaphylaxis cases evaluated over a 10-year period, reported that in some areas in the US, AGS was found to be the most common cause of anaphylaxis and represented the most striking difference over time (128). In a prospective study of patients with idiopathic anaphylaxis, 9% had IgE to α-Gal (129).

With regards to atopy as a risk factor for AGS, data are conflicting between different cohorts. We have noted that more than half of our Swedish AGS patients were atopic (122). Other European studies also found an association between atopy and sensitization to α-Gal, but in an AGS cohort from US such association was absent (34, 130, 131). Interestingly, a study on patients experiencing anaphylaxis upon the first dose of cetuximab found a strong association with atopic history (48). Furthermore, in our Swedish cohort, atopy increased the risk of experiencing anaphylactic symptoms in the respiratory system (122), but in the US cohort atopy was not associated with the severity of reactions (34). One reason for the discrepancy could be the definition of atopy.

Unlike most food allergies, where ingestion of small amounts of allergen elicits reactions, there is a wide interindividual variability in AGS with respect to eliciting dose of α-Gal, as well as type of food ingested (132). Some patients react only to foods containing high amounts of α-Gal, e.g. innards, while others may react to low amounts e.g. milk (40, 114, 133). Patients with AGS may not react with every ingestion of mammalian food (46, 118, 133) and the type and severity of the reaction may be different at different occasions. This may depend on the amount of α-Gal in the mammalian food, or the presence of co-factors such as consumption of alcohol, intake of non-steroidal anti-inflammatory drugs, physical exercise and infection that can increase intestinal absorption and lower the threshold dose (133). Wide interindividual variability is also present with respect to onset of symptoms. While symptoms mainly appear with a delay of at least 2 hours after ingestion of α-Gal containing foods, some AGS patients may experience symptom onset in less than 1 hour after innards consumption, or after as much as 11 hours after gelatin consumption (47, 133).

AGS patients often describe large local reactions at the site of tick bites. Most AGS patients also have IgE against tick extract (91, 134). Furthermore, recent tick bites appear to make patients more sensitive to prior tolerated exposures, or even lower the threshold for reactivity (135). It is well known that continuous tick bites contribute to a sustained and/or increased level of anti-Gal IgE in AGS patients, as well as in tick-bitten subjects (63, 136, 137). Conversely, AGS patients that do not experience further tick bites typically present decreased IgE levels to α-Gal over time (134, 136).

The implication of sensitization to α-Gal goes beyond allergic responses, as it may be a risk factor for coronary artery disease (CAD). Wilson et al. have shown that among 118 adult patients who underwent coronary characterization at the discretion of their cardiologist, 26% had IgE to α-Gal. The authors reported that sensitization to α-Gal was associated with an increased atheroma burden and plaques with more unstable features (138). Another larger cross-sectional study on more than 1000 individuals with suspected CAD, found that α-Gal sensitization was independently associated with noncalcified plaque burden and obstructive CAD (139). This association is only statistical so far and there are no mechanistic studies behind it. The proposed hypothesis is that in subjects who make IgE to α-Gal, dietary α-Gal-containing glycolipids carried by LDL induce low level of activation of mast cells residing within the atherosclerotic plaques, thus causing the chronic inflammation in the walls of coronary arteries (140).

## Prevalence of AGS

The association between tick bites and red meat allergy was described for the first time in Australia in 2007 (141). Two years later, Commins et al. reported a relationship between red meat allergy and IgE antibodies to α-Gal (1). Shortly after, AGS reports came from Spain, France, Sweden, Japan (33, 43, 142–144), and today AGS is recognized worldwide. Although it is evident that AGS is a growing health problem, there are no current estimates of global prevalence. A recent large study from the US analyzed data on α-Gal-specific IgE in serum samples from almost 300 000 subjects submitted to the commercial laboratory Viracor. The authors estimated that up to 450 000 persons in the United States might have been affected by AGS (3). An earlier report from Australia estimated that the prevalence of AGS in endemic regions of *I. holocyclus* ticks is one in every 550 people (61). An unselected cohort (n = 232) from central Virginia displayed a high prevalence of IgE to α-Gal (145). A study from Hamsten et al. investigated sensitization to α-Gal in a Swedish general population and found that as many as 10% (from a total of 143 healthy blood donors from the greater Stockholm area) had IgE antibodies to α-Gal (>0.1 kU_A_/L) (33). Similarly, general populations from sub-urban Copenhagen and north-western Spain displayed sensitization to α-Gal (>0.1 kU_A_/L) in 5.5% and 8.1%, respectively, and the sensitization was associated with tick bites (130). In addition, screening of two Swedish cohorts of tick bitten subjects (207 patients with Lyme disease from greater Stockholm area, and 148 patients with erythema migrans from South Sweden) showed a prevalence of 22% sensitization to α-Gal (33, 146). The frequency of sensitization to α-Gal has shown to be approximately 20 times higher in subjects living in a rural pre-Alps area of Italy with high exposure to ticks, than in subjects living in a nearby urban area (131). Additional evidence that a life-style with increased tick exposure is associated with sensitization to α-Gal, came from two European studies on forestry workers that found a sensitization frequency of 35% (n = 105/300), and 15% (n = 22/147) (147, 148). However, in these two cohorts only 1,7% and none suffered from AGS. Thus, sensitization to α-Gal does not necessarily imply having AGS as many sensitized subjects tolerate intake of mammalian meat.

Prevalence of α-Gal sensitization among young adults seems to be lower. In the large BAMSE cohort (n = 2201) of Swedish young adults from urban and sub-urban areas, approximately 6% were sensitized to α-Gal, but AGS was rare, only 0.1% (149). Although the majority had been tick bitten, the prevalence of sensitization increased with increasing number of tick bites. This study demonstrated that the association between tick bites and α-Gal sensitization is present already in young adults. Similarly, among 3000 young Americans, 6% were sensitized to α-Gal, and exposure to *A. americanum*, rural residence, and white race were identified as risk factors (150). The low prevalence of AGS found at young adulthood may be that the IgE levels to α-Gal are still low and repeated tick bites are required for symptoms to develop (149).

With climate change and global warming, tick populations are increasing, and their geographic range is expanding (151–155). Thus, it is likely that the incidence of AGS will be rising.

## Diagnosis

AGS is often under-/misdiagnosed due to the delayed spectrum of symptoms, as well as non-specific symptoms which may lead to a long-lasting search for a diagnosis (156). A study by Flaherty et al. aimed to determine the path to diagnosis experienced by AGS patients and found that only one fifth of patients received a diagnosis within their first year of symptoms, whereas the remaining approximately 80% received a diagnosis in an average of 7.1 years (157). Furthermore, in most cases the diagnosis appeared to be patient-driven (157). A new onset of reactions to red meat should alert clinicians to suspicion of AGS. Thus, there is a need for increased healthcare provider education and awareness of AGS to accelerate and improve the accuracy of AGS diagnoses, patient care, and understanding of the epidemiology of this emerging condition (158).

The diagnosis is based on the case history and IgE antibody levels to α-Gal. There are no established criteria for the level of α-Gal IgE that confirms an AGS diagnosis, but a cut-off >0.1 kU_A_/L as a positive test result has been used by most clinical authorities. IgE to bovine thyroglobulin (bTG) of more than 0.35 kU_A_/L has a reported specificity of 92.3% and sensitivity of 100% for diagnosis of AGS (159). IgE to bTG showed higher diagnostic value than IgE to beef, and significantly outperformed IgE to pork and lamb (159). Furthermore, IgE levels to α-Gal are greater than or equal to 2 kU_A_/L or more than 2% of the total IgE, makes the diagnosis very likely (115). The α-Gal IgE levels do not differ between patients experiencing an early (2 hours or less) versus a delayed reaction (more than 2 hours) (34, 122). Moreover, IgE binding to lactoferrin in AGS has been shown to be associated with a risk of having anaphylactic reactions to red meat (36). We have further shown that IgE levels to α-Gal and lactoferrin were significantly higher in patients with a history of allergic reactions to dairy, but receiver-operating characteristic curve analysis showed that the sensitivity and specificity were not sufficient to predict reactivity to dairy (40).

Common *in vivo* diagnostic tests, such as skin prick tests with meat extracts, have reported to be unreliable (1). However, prick-to-prick tests with raw innards have shown to have high sensitivity (160). If the above-mentioned tests are negative, despite a convincing history, basophil activation test (BAT) may be helpful. Mehlich et al. have shown that BAT can distinguish AGS patients from α-Gal sensitized individuals (99). We further evaluated BAT as a tool to discriminate between patients with severe i.e. anaphylactic reaction from non-anaphylactic patients, but did not find a positive association (161).

Food challenge is the golden standard in the diagnosis of food allergy, but due to the unpredictable nature of α-Gal food challenges, risks and benefits should be discussed with the patients (135). Finally, if the patient reports improvement of symptoms after mammalian meat avoidance, this supports the diagnosis (115).

## Management

Most AGS patients should be advised to avoid mammalian meat as well as organ meat and sausages. We have previously shown that the allergenicity of red meat proteins is preserved even upon different thermal cooking, thus AGS patients should avoid not only raw or medium rare meat, but also heat-treated meat (38).

Some patients need to avoid dairy products as well. We and others have recently reported that approximately 5 - 20% of AGS patients need avoidance of dairy products (40, 114, 115). However, approximately half of our AGS patients experienced allergic reactions to milk or dairy products at certain exposures only and that depended on the amount consumed, type of dairy products and co-factors (40). Patients experiencing reactions to dairy are more likely to report GI symptoms (40, 114). However, dairy products do not need to be avoided routinely, only if the patient continues to have symptoms despite strict avoidance of mammalian meat (115). A minor proportion of AGS patients need to avoid gelatin-containing food as well (115). An open question is whether consumption of small amounts of α-Gal could be protective and help regain tolerance to mammalian meat (162). Some clinicians encourage AGS patients to incorporate moderate amounts of dairy products in their diets unless they report adverse reactions following the ingestion of dairy (163). Patients who tolerate dairy, avoid further tick bites and have significant drops in IgE levels to α-Gal are more likely to successfully reintroduce mammalian food products into their diet if desired (163).

Some pharmaceuticals contain α-Gal and should be avoided or administered with caution in AGS patients (164). For instance, mammalian antibodies, such as cetuximab and antivenoms contain α-Gal and present a high risk for AGS patients. Drugs of porcine origin such as porcine pancreatic enzyme replacement contain α-Gal and may induce symptoms in some patients, while others demonstrate drug tolerance (135, 165). Gelatin in medical products (certain vaccines, plasma expanders, or gel-based capsules) may cause reactions in AGS patients (115). Heparin, used as an anti-coagulant, is a glycosaminoglycan isolated from porcine or bovine intestine and does not appear to carry α-Gal. However, there are case reports on anaphylactic reactions caused by heparin in AGS patients which could be due to contamination with α-Gal-carrying molecules (166, 167). Bioprosthetic heart valves can be from porcine or bovine sources, and patients with AGS undergoing bioprosthetic valve replacement are at the risk of developing a hypersensitivity reaction after surgery (168–170). Furthermore, products such as glycerin and magnesium stearate, that might hypothetically contain α-Gal need caution, though reactions to these ingredients have not been clearly demonstrated (171).

An important part of management of AGS is avoidance of tick bites. The patients should be informed about the relevance of tick bites on the onset of the disease and that further tick bites increase IgE levels to α-Gal, while avoidance of tick bites decreases their IgE levels to α-Gal. There are cases whose IgE levels to α-Gal became negative (or significantly dropped) after tick avoidance and they were able to reintroduce mammalian meat in their diet (115, 163, 172). AGS patients should wear long trousers and long-sleeved shirts in wooded and grassy areas and use insect repellents. Clothing and body should be checked for ticks after being outdoors.

There is no cure for AGS. Recently, a small oral immunotherapy (OIT) study for early and delayed-onset red meat allergy was reported (173). Five patients underwent an early OIT and seven a delayed protocol and the patients were followed for up to five years. All patients became tolerant to red meat after OIT, however three patients had to terminate OIT. One patient dropped out because of discontinuation of the maintenance regimen and two due to rare tick bites that acted as inducers of allergic reactions with concomitant elevation of the α-Gal IgE concentrations. The results of the study should be carefully interpreted in terms of few patients and performed in only one country. Larger patient cohorts from several countries are needed to further evaluate OIT.

## Concluding remarks

It has been more than a decade since the α-Gal epitope, a structural homolog of a blood group B antigen, was shown to be the cause of a global allergic disease, AGS. It is the first known allergy where a carbohydrate solely is the cause of IgE-mediated allergic reactions. The clinical significance of the α-Gal epitope led to the recent incorporation of glycan epitopes into the WHO/IUIS Allergen database. AGS represents a severe form of delayed allergy to mammalian meat and is an increasingly recognized public health issue. Most commonly, it develops in middle-aged patients who have previously tolerated mammalian food, but children can also be affected. Importantly, AGS is considered the first tick bite acquired allergic disease.

The knowledge of AGS has increased greatly during the last years. However, the impact of tick saliva on the development of AGS as well as the mechanisms behind the delayed reaction need to be elucidated. Moreover, the host characteristics that predispose to developing AGS are to be revealed. In addition, the relevance of α-Gal sensitization beyond allergic disease is a topic for further investigation. Currently there is no treatment option for AGS apart from avoidance of mammalian allergen sources. Therefore, there is a need for future research to better understand the immunological responses to the α-Gal epitope which will pave the way forward to the development of a safe and effective therapy for AGS.

## Author contributions

MP: Conceptualization, Writing – original draft, Writing – review & editing, Funding acquisition. JG: Conceptualization, Writing – original draft, Writing – review & editing. ME: Writing – review & editing. CH: Writing – review & editing. DA: Writing – original draft, Writing – review & editing, Funding acquisition. MvH: Conceptualization, Writing – original draft, Writing – review & editing, Funding acquisition.

## References

[1] Commins SP Satinover SM Hosen J Mozena J Borish L Lewis BD. Delayed anaphylaxis, angioedema, or urticaria after consumption of red meat in patients with IgE antibodies specific for galactose-alpha-1,3-galactose. J Allergy Clin Immunol (2009) 123(2):426–33. doi: 10.1016/j.jaci.2008.10.052 PMC3324851 19070355

[2] Van Nunen AS O’Connor KS Clarke LR Boyle XR Fernando SL. An association between tick bite reactions and red meat allergy in humans. Med J Aust (2009) 190(9):510–1. doi: 10.5694/j.1326-5377.2009.tb02533.x 19413526

[3] Thompson JM Carpenter A Kersh GJ Wachs T Commins SP Salzer JS. Geographic distribution of suspected alpha-gal syndrome cases — United States, January 2017–December 2022. Morbidity Mortality Weekly Rep (2023) 72(30):815–20. doi: 10.15585/mmwr.mm7230a2 PMC10390090 37498787

[4] Platts-Mills TA Hilger C Jappe U van Hage M Gadermaier G Spillner E. Carbohydrate epitopes currently recognized as targets for IgE antibodies. Allergy (2021) 76(8):2383–94. doi: 10.1111/all.14802 PMC8489568 33655520

[5] Aalberse RC Koshte V Clemens JG. Immunoglobulin E antibodies that crossreact with vegetable foods, pollen, and Hymenoptera venom. J Allergy Clin Immunol (1981) 68(5):356–64. doi: 10.1016/0091-6749(81)90133-0 7298999

[6] van Ree R. Clinical importance of cross-reactivity in food allergy. Curr Opin Allergy Clin Immunol (2004) 4(3):235–40. doi: 10.1097/00130832-200406000-00017 15126948

[7] Mari A. IgE to cross-reactive carbohydrate determinants: analysis of the distribution and appraisal of the in *vivo* and in *vitro* reactivity. Int Arch Allergy Immunol (2002) 129(4):286–95. doi: 10.1159/000067591 12483033

[8] Kaulfurst-Soboll H Mertens M Brehler R von Schaewen A. Reduction of cross-reactive carbohydrate determinants in plant foodstuff: elucidation of clinical relevance and implications for allergy diagnosis. PloS One (2011) 6(3):e17800. doi: 10.1371/journal.pone.0017800 21423762 PMC3056789

[9] Holzweber F Svehla E Fellner W Dalik T Stubler S Hemmer W. Inhibition of IgE binding to cross-reactive carbohydrate determinants enhances diagnostic selectivity. Allergy (2013) 68(10):1269–77. doi: 10.1111/all.12229 PMC4223978 24107260

[10] Foetisch K Westphal S Lauer I Retzek M Altmann F Kolarich D. Biological activity of IgE specific for cross-reactive carbohydrate determinants. J Allergy Clin Immunol (2003) 111(4):889–96. doi: 10.1067/mai.2003.173 12704374

[11] Paulus KE Mahler V Pabst M Kogel KH Altmann F Sonnewald U. Silencing beta1,2-xylosyltransferase in Transgenic Tomato Fruits Reveals xylose as Constitutive Component of Ige-Binding Epitopes. Front Plant Sci (2011) 2:42. doi: 10.3389/fpls.2011.00042 22639593 PMC3355614

[12] Bublin M Radauer C Wilson IB Kraft D Scheiner O Breiteneder H. Cross-reactive N-glycans of Api g 5, a high molecular weight glycoprotein allergen from celery, are required for immunoglobulin E binding and activation of effector cells from allergic patients. FASEB J (2003) 17(12):1697–9. doi: 10.1096/fj.02-0872fje 12958180

[13] Gattinger P Bidovec-Stojkovic U Zidarn M Korosec P Valenta R Mittermann I. Glycosylation enhances allergenic activity of major bee venom allergen Api m 1 by adding IgE epitopes. J Allergy Clin Immunol (2021) 147(4):1502–4. doi: 10.1016/j.jaci.2020.10.002 33045279

[14] Li M GustChina A Glesner J Wunschmann S Vailes LD Chapman MD. Carbohydrates contribute to the interactions between cockroach allergen Bla g 2 and a monoclonal antibody. J Immunol (2011) 186(1):333–40. doi: 10.4049/jimmunol.1002318 PMC3099132 21123808

[15] Do DC Yang S Yao X Hamilton RG Schroeder JT Gao P. N-glycan in cockroach allergen regulates human basophil function. Immun Inflammation Dis (2017) 5(4):386–99. doi: 10.1002/iid3.145 PMC5691304 28474843

[16] Commins SP Platts-Mills TA. Allergenicity of carbohydrates and their role in anaphylactic events. Curr Allergy Asthma Rep (2010) 10(1):29–33. doi: 10.1007/s11882-009-0079-1 20425511 PMC3057034

[17] Galili U BSS Kobrin E Stults CL Macher BA. Man, apes, and Old World monkeys differ from other mammals in the expression of alpha-galactosyl epitopes on nucleated cells. J Biol Chem (1988) 263(33):17755–62. doi: 10.1016/S0021-9258(19)77900-9 2460463

[18] Blanken WM Van den Eijnden DH. Biosynthesis of terminal Gal alpha 1-3Gal beta 1-4GlcNAc-R oligosaccharide sequences on glycoconjugates. Purification and acceptor specificity of a UDP-Gal:N-acetyllactosaminide alpha 1-3-galactosyltransferase from calf thymus. J Biol Chem (1985) 260(24):12927–34. doi: 10.1016/S0021-9258(17)38814-2 3932335

[19] Hamadeh RM Galili U Zhou P Griffiss JM. Anti-alpha-galactosyl immunoglobulin A (IgA), IgG, and IgM in human secretions. Clin Diagn Lab Immunol (1995) 2(2):125–31. doi: 10.1128/cdli.2.2.125-131.1995 PMC170114 7697518

[20] Montassier E Al-Ghalith GA Mathe C Le Bastard Q Douillard V Garnier A. Distribution of bacterial alpha1,3-galactosyltransferase genes in the human gut microbiome. Front Immunol (2019) 10:3000. doi: 10.3389/fimmu.2019.03000 31998300 PMC6970434

[21] Galili U Mandrell RE Hamadeh RM Shohet SB Griffiss JM. Interaction between human natural anti-alpha-galactosyl immunoglobulin G and bacteria of the human flora. Infect Immun (1988) 56(7):1730–7. doi: 10.1128/iai.56.7.1730-1737.1988 PMC259469 3290105

[22] Barreau N Blancho G Boulet C Martineau A Vusio P Liaigre J. Natural anti-Gal antibodies constitute 0.2% of intravenous immunoglobulin and are equally retained on a synthetic disaccharide column or on an immobilized natural glycoprotein. Transplant Proc (2000) 32(5):882–3. doi: 10.1016/s0041-1345(00)01023-x 10936257

[23] Galili U. Anti-Gal: an abundant human natural antibody of multiple pathogeneses and clinical benefits. Immunology (2013) 140(1):1–11. doi: 10.1111/imm.12110 PMC3809700 23578170

[24] Wang L Radic MZ Galili U. Human anti-Gal heavy chain genes. Preferential use of VH3 and the presence of somatic mutations. J Immunol (1995) 155(3):1276–85. 7543518

[25] Kearns-Jonker M Swensson J Ghiuzeli C Chu W Osame Y Starnes V. The human antibody response to porcine xenoantigens is encoded by IGHV3-11 and IGHV3-74 IgVH germline progenitors. J Immunol (1999) 163(8):4399–412. 10510381

[26] Langley DB Schofield P Nevoltris D Jackson J Jackson KJL Peters TJ. Genetic and structural basis of the human anti-alpha-galactosyl antibody response. Proc Natl Acad Sci USA (2022) 119(28):e2123212119. doi: 10.1073/pnas.2123212119 35867757 PMC9282431

[27] Hamanova M Chmelikova M Nentwich I Thon V Lokaj J. Anti-Gal IgM, IgA and IgG natural antibodies in childhood. Immunol Lett (2015) 164(1):40–3. doi: 10.1016/j.imlet.2015.02.001 25684746

[28] Galili U. Human natural antibodies to mammalian carbohydrate antigens as unsung heroes protecting against past, present, and future viral infections. Antibodies (Basel) (2020) 9(2):25. doi: 10.3390/antib9020025 32580274 PMC7344964

[29] Galili U. The alpha-gal epitope and the anti-Gal antibody in xenotransplantation and in cancer immunotherapy. Immunol Cell Biol (2005) 83(6):674–86. doi: 10.1111/j.1440-1711.2005.01366.x 16266320

[30] Galili U Buehler J Shohet SB Macher BA. The human natural anti-Gal IgG. III. The subtlety of immune tolerance in man as demonstrated by crossreactivity between natural anti-Gal and anti-B antibodies. J Exp Med (1987) 165(3):693–704. doi: 10.1084/jem.165.3.693 2434599 PMC2188289

[31] Apostolovic D Rodrigues R Thomas P Starkhammar M Hamsten C van Hage M. Immunoprofile of alpha-Gal- and B-antigen-specific responses differentiates red meat-allergic patients from healthy individuals. Allergy (2018) 73(7):1525–31. doi: 10.1111/all.13400 29319188

[32] Rispens T Derksen NI Commins SP Platts-Mills TA Aalberse RC. IgE production to alpha-gal is accompanied by elevated levels of specific IgG1 antibodies and low amounts of IgE to blood group B. PloS One (2013) 8(2):e55566. doi: 10.1371/journal.pone.0055566 23390540 PMC3563531

[33] Hamsten C Tran TAT Starkhammar M Brauner A Commins SP Platts-Mills TAE. Red meat allergy in Sweden: association with tick sensitization and B-negative blood groups. J Allergy Clin Immunol (2013) 132(6):1431–4. doi: 10.1016/j.jaci.2013.07.050 PMC4036066 24094548

[34] Wilson JM Schuyler AJ Workman L Gupta M James HR Posthumus J. Investigation into the alpha-Gal Syndrome: Characteristics of 261 Children and Adults Reporting Red Meat Allergy. J Allergy Clin Immunol Pract (2019) 7(7):2348–58 e4. doi: 10.1016/j.jaip.2019.03.031 30940532 PMC6993919

[35] Apostolovic D Tran TA Sanchez-Vidaurre S Cirkovic Velickovic T Starkhammar M Hamsten C. Red meat allergic patients have a selective IgE response to the alpha-Gal glycan. Allergy (2015) 70(11):1497–500. doi: 10.1111/all.12695 26190542

[36] Kiewiet MBG Grundstrom J Apostolovic D Andersson M Borres MP Hamsten C. Elucidating the alpha-Gal syndrome at the molecular allergen level. Allergy (2021) 76(5):1576–8. doi: 10.1111/all.14660 PMC8246984 33206401

[37] Takahashi H Chinuki Y Tanaka A Morita E. Laminin gamma-1 and collagen alpha-1 (VI) chain are galactose-alpha-1,3-galactose-bound allergens in beef. Allergy (2014) 69(2):199–207. doi: 10.1111/all.12302 24180678

[38] Apostolovic D Tran TA Hamsten C Starkhammar M Cirkovic Velickovic T van Hage M. Immunoproteomics of processed beef proteins reveal novel galactose-alpha-1,3-galactose-containing allergens. Allergy (2014) 69(10):1308–15. doi: 10.1111/all.12462 24942937

[39] Kollmann D Nagl B Ebner C Emminger W Wohrl S Kitzmuller C. The quantity and quality of alpha-gal-specific antibodies differ in individuals with and without delayed red meat allergy. Allergy (2017) 72(2):266–73. doi: 10.1111/all.12948 PMC5244683 27261450

[40] Perusko M Apostolovic D Kiewiet MBG Grundstrom J Hamsten C Starkhammar M. Bovine gamma-globulin, lactoferrin, and lactoperoxidase are relevant bovine milk allergens in patients with alpha-Gal syndrome. Allergy (2021) 76(12):3766–75. doi: 10.1111/all.14889 33938008

[41] Thall A Galili U. Distribution of Gal alpha 1—-3Gal beta 1—-4GlcNAc residues on secreted mammalian glycoproteins (thyroglobulin, fibrinogen, and immunoglobulin G) as measured by a sensitive solid-phase radioimmunoassay. Biochemistry (1990) 29(16):3959–65. doi: 10.1021/bi00468a024 2354167

[42] German-Sanchez A Alonso-Llamazares A Latorre-Ibanez MD Bartolome-Zavala B Antepara-Ercoreca I. Sheep cheese allergy in Alpha-gal Syndrome. J Investig Allergol Clin Immunol (2023) 33(6):491–3. doi: 10.18176/jiaci.0897 36811837

[43] Morisset M Richard C Astier C Jacquenet S Croizier A Beaudouin E. Anaphylaxis to pork kidney is related to IgE antibodies specific for galactose-alpha-1,3-galactose. Allergy (2012) 67(5):699–704. doi: 10.1111/j.1398-9995.2012.02799.x 22494361

[44] Jappe U Minge S Kreft B Ludwig A Przybilla B Walker A. Meat allergy associated with galactosyl-alpha-(1,3)-galactose (alpha-Gal)-Closing diagnostic gaps by anti-alpha-Gal IgE immune profiling. Allergy (2018) 73(1):93–105. doi: 10.1111/all.13238 28670695 PMC7254904

[45] Hilger C Fischer J Swiontek K Hentges F Lehners C Eberlein B. Two galactose-alpha-1,3-galactose carrying peptidases from pork kidney mediate anaphylactogenic responses in delayed meat allergy. Allergy (2016) 71(5):711–9. doi: 10.1111/all.12835 26728983

[46] Mullins RJ James H Platts-Mills TA Commins S. Relationship between red meat allergy and sensitization to gelatin and galactose-alpha-1,3-galactose. J Allergy Clin Immunol (2012) 129(5):1334–42 e1. doi: 10.1016/j.jaci.2012.02.038 22480538 PMC3340561

[47] Caponetto P Fischer J Biedermann T. Gelatin-containing sweets can elicit anaphylaxis in a patient with sensitization to galactose-alpha-1,3-galactose. J Allergy Clin Immunol Pract (2013) 1(3):302–3. doi: 10.1016/j.jaip.2013.01.007 24565491

[48] O'Neil BH Allen R Spigel DR Stinchcombe TE Moore DT Berlin JD. High incidence of cetuximab-related infusion reactions in Tennessee and North Carolina and the association with atopic history. J Clin Oncol (2007) 25(24):3644–8. doi: 10.1200/JCO.2007.11.7812 17704414

[49] Chung CH Mirakhur B Chan E Le QT Berlin J Morse M. Cetuximab-induced anaphylaxis and IgE specific for galactose-alpha-1,3-galactose. N Engl J Med (2008) 358(11):1109–17. doi: 10.1056/NEJMoa074943 PMC2361129 18337601

[50] Fischer J Eberlein B Hilger C Eyer F Eyerich S Ollert M. Alpha-gal is a possible target of IgE-mediated reactivity to antivenom. Allergy (2017) 72(5):764–71. doi: 10.1111/all.13073 27775867

[51] Rizer J Brill K Charlton N King J. Acute hypersensitivity reaction to Crotalidae polyvalent immune Fab (CroFab) as initial presentation of galactose-alpha-1,3-galactose (alpha-gal) allergy. Clin Toxicol (Phila) (2017) 55(7):668–9. doi: 10.1080/15563650.2017.1313981 28443380

[52] Straesser M Keshavarz B Borish L Khokhar D Holian A Charlton NP. Alpha-Gal on Crotalidae-polyvalent Fab antivenom (CroFab): Investigating the relevance to immediate hypersensitivity reactions. J Allergy Clin Immunol Pract (2021) 9(2):1015–7 e1. doi: 10.1016/j.jaip.2020.10.026 33122101 PMC7870545

[53] Stone CA Jr. Commins SP Choudhary S Vethody C Heavrin JL Wingerter J. Anaphylaxis after vaccination in a pediatric patient: further implicating alpha-gal allergy. J Allergy Clin Immunol Pract (2019) 7(1):322–4 e2. doi: 10.1016/j.jaip.2018.06.005 29913263 PMC6295282

[54] Stone CA Jr. Hemler JA Commins SP Schuyler AJ Phillips EJ Peebles RS Jr.. Anaphylaxis after zoster vaccine: Implicating alpha-gal allergy as a possible mechanism. J Allergy Clin Immunol (2017) 139(5):1710–3 e2. doi: 10.1016/j.jaci.2016.10.037 27986511 PMC5420485

[55] Schmidle P Mehlich J Brockow K Darsow U Biedermann T Eberlein B. Gelatin-containing vaccines for varicella, zoster, measles, mumps, and rubella induce basophil activation in patients with alpha-gal syndrome. Int Arch Allergy Immunol (2021) 182(8):716–22. doi: 10.1159/000514263 33735861

[56] Serrier J Khoy K Ollivier Y Gervais R Le Moel G Lafosse M. Recurrent anaphylaxis to a gelatin-based colloid plasma substitute and to cetuximab following sensitisation to galactose-alpha-1,3-galactose. Br J Anaesth (2021) 126(6):e200–e2. doi: 10.1016/j.bja.2021.02.013 33810867

[57] Apostolovic D Mihailovic J Commins SP Wijnveld M Kazimirova M Starkhammar M. Allergenomics of the tick *Ixodes ricinus* reveals important alpha-Gal-carrying IgE-binding proteins in red meat allergy. Allergy (2020) 75(1):217–20. doi: 10.1111/all.13978 PMC8304496 31301243

[58] Crispell G Commins SP Archer-Hartman SA Choudhary S Dharmarajan G Azadi P. Discovery of alpha-gal-containing antigens in North American tick species believed to induce red meat allergy. Front Immunol (2019) 10. doi: 10.3389/fimmu.2019.01056 PMC6533943 31156631

[59] Commins SP James HR Kelly LA Pochan SL Workman LJ Perzanowski MS. The relevance of tick bites to the production of IgE antibodies to the mammalian oligosaccharide galactose-alpha-1,3-galactose. J Allergy Clin Immunol (2011) 127(5):1286–93 e6. doi: 10.1016/j.jaci.2011.02.019 21453959 PMC3085643

[60] Chinuki Y Ishiwata K Yamaji K Takahashi H Morita E. Haemaphysalis longicornis tick bites are a possible cause of red meat allergy in Japan. Allergy (2016) 71(3):421–5. doi: 10.1111/all.12804 26551325

[61] van Nunen S. Tick-induced allergies: mammalian meat allergy, tick anaphylaxis and their significance. Asia Pac Allergy (2015) 5(1):3–16. doi: 10.5415/apallergy.2015.5.1.3 25653915 PMC4313755

[62] Araujo RN Franco PF Rodrigues H Santos LCB McKay CS Sanhueza CA. *Amblyomma sculptum* tick saliva: alpha-Gal identification, antibody response and possible association with red meat allergy in Brazil. Int J Parasitol (2016) 46(3):213–20. doi: 10.1016/j.ijpara.2015.12.005 PMC5523130 26812026

[63] Hashizume H Fujiyama T Umayahara T Kageyama R Walls AF Satoh T. Repeated *Amblyomma testudinarium* tick bites are associated with increased galactose-alpha-1,3-galactose carbohydrate IgE antibody levels: A retrospective cohort study in a single institution. J Am Acad Dermatol (2018) 78(6):1135–41 e3. doi: 10.1016/j.jaad.2017.12.028 29273488

[64] Hamsten C Starkhammar M Tran TA Johansson M Bengtsson U Ahlen G. Identification of galactose-alpha-1,3-galactose in the gastrointestinal tract of the tick *Ixodes ricinus*; possible relationship with red meat allergy. Allergy (2013) 68(4):549–52. doi: 10.1111/all.12128 23414348

[65] Cabezas-Cruz A Espinosa PJ Alberdi P Simo L Valdes JJ Mateos-Hernandez L. Tick galactosyltransferases are involved in alpha-Gal synthesis and play a role during *Anaplasma phagocytophilum* infection and *Ixodes scapularis* tick vector development. Sci Rep (2018) 8(1):14224. doi: 10.1038/s41598-018-32664-z 30242261 PMC6154994

[66] Platts-Mills TAE Commins SP Biedermann T van Hage M Levin M Beck LA. On the cause and consequences of IgE to galactose-alpha-1,3-galactose: A report from the National Institute of Allergy and Infectious Diseases Workshop on Understanding IgE-Mediated Mammalian Meat Allergy. J Allergy Clin Immunol (2020) 145(4):1061–71. doi: 10.1016/j.jaci.2020.01.047 PMC7301618 32057766

[67] Fischer J Riel S Fehrenbacher B Frank A Schaller M Biedermann T. Spatial distribution of alpha-gal in *Ixodes ricinus* - A histological study. Ticks Tick Borne Dis (2020) 11(5):101506. doi: 10.1016/j.ttbdis.2020.101506 32723636

[68] Jasinskas A Barbour AG. The Fc fragment mediates the uptake of immunoglobulin G from the midgut to hemolymph in the ixodid tick *amblyomma americanum* (Acari: ixodidae). J Med Entomol (2005) 42(3):359–66. doi: 10.1093/jmedent/42.3.359 15962788

[69] Platts-Mills T. Sensitisation of Forest workers to the oligosaccharide galactose alpha-1, 3-galactose (alpha-gal) is strongly associated with tick bites but not with evidence of tick borne infections. Infect Dis (Lond) (2022) 54(8):580–2. doi: 10.1080/23744235.2022.2057584 35382674

[70] Rutkowski K Sowa P Mroczko B Pancewicz S Rutkowski R Czupryna P. Sensitisation and allergic reactions to alpha-1,3-galactose in Podlasie, Poland, an area endemic for tick-borne infections. Infect Dis (Lond) (2022) 54(8):572–9. doi: 10.1080/23744235.2022.2057583 35382677

[71] Benders-Guedj M Koberle M Hofmann H Biedermann T Darsow U. High-risk groups for alpha-gal sensitization. Allergol Select (2023) 7:140–8. doi: 10.5414/ALX02424E PMC10495941 37705677

[72] Stoltz LP Cristiano LM Dowling APG Wilson JM Platts-Mills TAE Traister RS. Could chiggers be contributing to the prevalence of galactose-alpha-1,3-galactose sensitization and mammalian meat allergy? J Allergy Clin Immunol Pract (2019) 7(2):664–6. doi: 10.1016/j.jaip.2018.07.014 PMC6549691 30053595

[73] Choudhary S Jerath MR Commins SP. Venom allergy is increased in alpha-gal allergy: shared environmental or immunologic factors? J Allergy Clin Immunol (2018) 141(2):AB199. doi: 10.1016/j.jaci.2017.12.631

[74] Kutlu A Unal D. Mammalian meat allergy accompanied by venom allergy: A review of 12 cases. Iran J Allergy Asthma Immunol (2019) 18(5):584–8. doi: 10.18502/ijaai.v18i5.1928 32245302

[75] Kiewiet MBG Perusko M Grundstrom J Hamsten C Starkhammar M Apostolovic D. Cross-reactivity between tick and wasp venom can contribute to frequent wasp sensitization in patients with the alpha-Gal syndrome. Clin Transl Allergy (2022) 12(1):e12113. doi: 10.1002/clt2.12113 35070272 PMC8762686

[76] Skallova A Iezzi G Ampenberger F Kopf M Kopecky J. Tick saliva inhibits dendritic cell migration, maturation, and function while promoting development of Th2 responses. J Immunol (2008) 180(9):6186–92. doi: 10.4049/jimmunol.180.9.6186 18424740

[77] Cavassani KA Aliberti JC Dias AR Silva JS Ferreira BR. Tick saliva inhibits differentiation, maturation and function of murine bone-marrow-derived dendritic cells. Immunology (2005) 114(2):235–45. doi: 10.1111/j.1365-2567.2004.02079.x PMC1782083 15667568

[78] Mejri N Brossard M. Splenic dendritic cells pulsed with *Ixodes ricinus* tick saliva prime naive CD4+T to induce Th2 cell differentiation *in vitro* and *in vivo* . Int Immunol (2007) 19(4):535–43. doi: 10.1093/intimm/dxm019 17344202

[79] Carvalho-Costa TM Mendes MT da Silva MV da Costa TA Tiburcio MG Anhe AC. Immunosuppressive effects of Amblyomma cajennense tick saliva on murine bone marrow-derived dendritic cells. Parasit Vectors (2015) 8:22. doi: 10.1186/s13071-015-0634-7 25586117 PMC4304185

[80] Xu Z Lin Z Wei N Di Q Cao J Zhou Y. Immunomodulatory effects of Rhipicephalus haemaphysaloides serpin RHS2 on host immune responses. Parasit Vectors (2019) 12(1):341. doi: 10.1186/s13071-019-3607-4 31296257 PMC6624921

[81] Sajiki Y Konnai S Ochi A Okagawa T Githaka N Isezaki M. Immunosuppressive effects of sialostatin L1 and L2 isolated from the taiga tick Ixodes persulcatus Schulze. Ticks Tick Borne Dis (2020) 11(2):101332. doi: 10.1016/j.ttbdis.2019.101332 31734217

[82] Sa-Nunes A Oliveira CJF. Dendritic cells as a disputed fortress on the tick-host battlefield. Trends Parasitol (2021) 37(4):340–54. doi: 10.1016/j.pt.2020.11.004 33303363

[83] Glatz M Means T Haas J Steere AC Mullegger RR. Characterization of the early local immune response to *Ixodes ricinus* tick bites in human skin. Exp Dermatol (2017) 26(3):263–9. doi: 10.1111/exd.13207 PMC5342933 27623398

[84] Krause PJ Grant-Kels JM Tahan SR Dardick KR Alarcon-Chaidez F Bouchard K. Dermatologic changes induced by repeated *Ixodes scapularis* bites and implications for prevention of tick-borne infection. Vector Borne Zoonotic Dis (2009) 9(6):603–10. doi: 10.1089/vbz.2008.0091 PMC2883515 19196014

[85] Kageyama R Fujiyama T Satoh T Keneko Y Kitano S Tokura Y. The contribution made by skin-infiltrating basophils to the development of alpha-gal syndrome. Allergy (2019) 74(9):1805–7. doi: 10.1111/all.13794 30903699

[86] Kovář L Kopecký J Říhová B. Salivary gland extract from *ixodes ricinus* tick polarizes the cytokine profile toward Th2 and suppresses proliferation of T lymphocytes in human pbmc culture. J Parasitology (2001) 87(6):1342–8. doi: 10.1645/0022-3395(2001)087[1342:SGEFIR]2.0.CO;2 11780819

[87] Thall AD Maly P Lowe JB. Oocyte Gal alpha 1,3Gal epitopes implicated in sperm adhesion to the zona pellucida glycoprotein ZP3 are not required for fertilization in the mouse. J Biol Chem (1995) 270(37):21437–40. doi: 10.1074/jbc.270.37.21437 7545161

[88] Tanemura M Galili U. T cells interacting with the alpha-Gal epitope: studies in alpha1, 3Galactosyltransferase knock-out mice. Transplant Proc (2000) 32(5):921–3. doi: 10.1016/S0041-1345(00)01037-X 10936273

[89] Cretin N Bracy J Hanson K Iacomini J. The role of T cell help in the production of antibodies specific for Gal alpha 1-3Gal. J Immunol (2002) 168(3):1479–83. doi: 10.4049/jimmunol.168.3.1479 11801692

[90] Lin YJ Hara H Tai HC Long C Tokita D Yeh P. Suppressive efficacy and proliferative capacity of human regulatory T cells in allogeneic and xenogeneic responses. Transplantation (2008) 86(10):1452–62. doi: 10.1097/TP.0b013e318188acb0 PMC2629735 19034017

[91] Apostolovic D Grundstrom J Kiewiet MBG Perusko M Hamsten C Starkhammar M. Th2-skewed T cells correlate with B cell response to alpha-Gal and tick antigens in alpha-Gal syndrome. J Clin Invest (2023) 133(6):e158357. doi: 10.1172/JCI158357 36701195 PMC10014093

[92] Hoof I Schulten V Layhadi JA Stranzl T Christensen LH Herrera de la Mata S. Allergen-specific IgG(+) memory B cells are temporally linked to IgE memory responses. J Allergy Clin Immunol (2020) 146(1):180–91. doi: 10.1016/j.jaci.2019.11.046 PMC7860973 31883847

[93] Aranda CJ Gonzalez-Kozlova E Saunders SP Fernandes-Braga W Ota M Narayanan S. IgG memory B cells expressing IL4R and FCER2 are associated with atopic diseases. Allergy (2023) 78(3):752–66. doi: 10.1111/all.15601 PMC9991991 36445014

[94] Ota M Hoehn KB Ota T Aranda CJ Friedman S Braga WF. The memory of pathogenic IgE is contained within CD23 (+) IgG1 (+) memory B cells poised to switch to IgE in food allergy. bioRxiv (2023). doi: 10.1101/2023.01.25.525506

[95] Koenig JFE Knudsen NPH Phelps A Bruton K Hoof I Lund G. A Distinct Phenotype of Polarized Memory B cell holds IgE Memory. BioRxiv (2023). doi: 10.1101/2023.01.25.525495

[96] Berkowska MA Heeringa JJ Hajdarbegovic E van der Burg M Thio HB van Hagen PM. Human IgE(+) B cells are derived from T cell-dependent and T cell-independent pathways. J Allergy Clin Immunol (2014) 134(3):688–97 e6. doi: 10.1016/j.jaci.2014.03.036 24835500

[97] Looney TJ Lee JY Roskin KM Hoh RA King J Glanville J. Human B-cell isotype switching origins of IgE. J Allergy Clin Immunol (2016) 137(2):579–86 e7. doi: 10.1016/j.jaci.2015.07.014 26309181 PMC4747810

[98] Roman-Carrasco P Hemmer W Klug C Friedrich A Stoll P Focke-Tejkl M. Individuals with IgE antibodies to alpha-Gal and CCD show specific IgG subclass responses different from subjects non-sensitized to oligosaccharides. Clin Exp Allergy (2020) 50(9):1107–10. doi: 10.1111/cea.13695 PMC7540519 32578253

[99] Mehlich J Fischer J Hilger C Swiontek K Morisset M Codreanu-Morel F. The basophil activation test differentiates between patients with alpha-gal syndrome and asymptomatic alpha-gal sensitization. J Allergy Clin Immunol (2019) 143(1):182–9. doi: 10.1016/j.jaci.2018.06.049 30125663

[100] Iweala OI Choudhary SK Addison CT Batty CJ Kapita CM Amelio C. Glycolipid-mediated basophil activation in alpha-gal allergy. J Allergy Clin Immunol (2020) 146(2):450–2. doi: 10.1016/j.jaci.2020.02.006 PMC7670280 32088306

[101] Commins SP James HR Stevens W Pochan SL Land MH King C. Delayed clinical and ex vivo response to mammalian meat in patients with IgE to galactose-alpha-1,3-galactose. J Allergy Clin Immunol (2014) 134(1):108–15. doi: 10.1016/j.jaci.2014.01.024 PMC4125475 24656556

[102] Wilson DH Platts-Mills T. The oligosaccharide galactose-α-1,3-galactose and the α-gal syndrome: insights from an epitope that is causal in immunoglobulin E-mediated immediate and delayed anaphylaxis. Allergy Immunol (2018) 3(1):89–98. doi: 10.33590/emjallergyimmunol/10310729

[103] Eller E Stahl Skov P Baumann K Hilger C Ollert M Bindslev-Jensen C. Delayed reaction in alpha-gal allergy is reflected in serum levels after ingestion of pork kidney, and absorption is dependent on food processing. Clin Exp Allergy (2022) 52(1):197–200. doi: 10.1111/cea.14054 34779547

[104] Labbe SM Grenier-Larouche T Croteau E Normand-Lauziere F Frisch F Ouellet R. Organ-specific dietary fatty acid uptake in humans using positron emission tomography coupled to computed tomography. Am J Physiol Endocrinol Metab (2011) 300(3):E445–53. doi: 10.1152/ajpendo.00579.2010 21098737

[105] Steinke JW Pochan SL James HR Platts-Mills TAE Commins SP. Altered metabolic profile in patients with IgE to galactose-alpha-1,3-galactose following in *vivo* food challenge. J Allergy Clin Immunol (2016) 138(5):1465–7 e8. doi: 10.1016/j.jaci.2016.05.021 27448448 PMC5099113

[106] Roman-Carrasco P Lieder B Somoza V Ponce M Szepfalusi Z Martin D. Only alpha-Gal bound to lipids, but not to proteins, is transported across enterocytes as an IgE-reactive molecule that can induce effector cell activation. Allergy (2019) 74(10):1956–68. doi: 10.1111/all.13873 PMC6852507 31102539

[107] Iweala O Brennan PJ Commins SP. Serum igE specific for alpha-gal sugar moiety can bind glycolipid. J Allergy Clin Immunol (2017) 139(2):AB88. doi: 10.1016/j.jaci.2016.12.237

[108] Bellutti Enders F Elkuch M Worner A Scherer Hofmeier K Hartmann K. Alpha-gal syndrome initially misdiagnosed as chronic spontaneous urticaria in a pediatric patient: a case report and review of the literature. J Med Case Rep (2023) 17(1):6. doi: 10.1186/s13256-022-03718-8 36611183 PMC9826568

[109] Chakrapani N Fischer J Swiontek K Codreanu-Morel F Hannachi F Morisset M. alpha-Gal present on both glycolipids and glycoproteins contributes to immune response in meat-allergic patients. J Allergy Clin Immunol (2022) 150(2):396–405 e11. doi: 10.1016/j.jaci.2022.02.030 35459547

[110] Krstic Ristivojevic M Grundstrom J Apostolovic D Radomirovic M Jovanovic V Radoi V. Alpha-gal on the protein surface hampers transcytosis through the caco-2 monolayer. Int J Mol Sci (2020) 21(16):5742. doi: 10.3390/ijms21165742 32796496 PMC7461108

[111] Cianferoni A. Non-igE-mediated anaphylaxis. J Allergy Clin Immunol (2021) 147(4):1123–31. doi: 10.1016/j.jaci.2021.02.012 33832694

[112] Caslin HL Kiwanuka KN Haque TT Taruselli MT MacKnight HP Paranjape A. Controlling mast cell activation and homeostasis: work influenced by bill paul that continues today. Front Immunol (2018) 9:868. doi: 10.3389/fimmu.2018.00868 29755466 PMC5932183

[113] Elieh Ali Komi D Shafaghat F Kovanen PT Meri S. Mast cells and complement system: Ancient interactions between components of innate immunity. Allergy (2020) 75(11):2818–28. doi: 10.1111/all.14413 32446274

[114] Binder AM Cherry-Brown D Biggerstaff BJ Jones ES Amelio CL Beard CB. Clinical and laboratory features of patients diagnosed with alpha-gal syndrome-2010-2019. Allergy (2023) 78(2):477–87. doi: 10.1111/all.15539 PMC10092820 36178236

[115] Platts-Mills TAE Li RC Keshavarz B Smith AR Wilson JM. Diagnosis and management of patients with the alpha-gal syndrome. J Allergy Clin Immunol Pract (2020) 8(1):15–23 e1. doi: 10.1016/j.jaip.2019.09.017 31568928 PMC6980324

[116] Boyce RM Schulz A Mansour O Giandomenico D Farel CE Commins SP. Alpha-gal syndrome in the infectious diseases clinic: A series of 5 cases in central North Carolina. Open Forum Infect Dis (2022) 9(12):ofac663. doi: 10.1093/ofid/ofac663 36582771 PMC9795474

[117] Young I Prematunge C Pussegoda K Corrin T Waddell L. Tick exposures and alpha-gal syndrome: A systematic review of the evidence. Ticks Tick Borne Dis (2021) 12(3):101674. doi: 10.1016/j.ttbdis.2021.101674 33529984

[118] Kennedy JL Stallings AP Platts-Mills TA Oliveira WM Workman L James HR. Galactose-alpha-1,3-galactose and delayed anaphylaxis, angioedema, and urticaria in children. Pediatrics (2013) 131(5):e1545–52. doi: 10.1542/peds.2012-2585 PMC3639458 23569097

[119] Mabelane T Basera W Botha M Thomas HF Ramjith J Levin ME. Predictive values of alpha-gal IgE levels and alpha-gal IgE: Total IgE ratio and oral food challenge-proven meat allergy in a population with a high prevalence of reported red meat allergy. Pediatr Allergy Immunol (2018) 29(8):841–9. doi: 10.1111/pai.12969 30144162

[120] Weyand CM Goronzy JJ. Aging of the immune system. Mechanisms and therapeutic targets. Ann Am Thorac Soc (2016) 13 Suppl 5(Suppl 5):S422–S8. doi: 10.1513/AnnalsATS.201602-095AW PMC5291468 28005419

[121] Simon AK Hollander GA McMichael A. Evolution of the immune system in humans from infancy to old age. Proc Biol Sci (2015) 282(1821):20143085. doi: 10.1098/rspb.2014.3085 26702035 PMC4707740

[122] Kiewiet MBG Apostolovic D Starkhammar M Grundstrom J Hamsten C van Hage M. Clinical and serological characterization of the α-gal syndrome-importance of atopy for symptom severity in a European cohort. J Allergy Clin Immunol Pract (2020) 8(6):2027–34. doi: 10.1016/j.jaip.2020.02.016 32142962

[123] McGill SK Levin ME Shaheen NJ Cotton CC Platts-Mills TA Commins SP. Gastrointestinal-isolated distress is common in alpha-gal allergic patients on mammalian meat challenge. J Clin Gastroenterol (2023) 58(1):80–4. doi: 10.1097/MCG.0000000000001827 PMC10314969 36728603

[124] Glynn D Halma J Welch H Shakhnovich V Friesen C. Nonanaphylactic variant of alpha-gal syndrome as an etiology for chronic gastrointestinal symptoms in children. J Pediatr (2023) 259:113486. doi: 10.1016/j.jpeds.2023.113486 37201681

[125] Pollack K Zlotoff BJ Borish LC Commins SP Platts-Mills TAE Wilson JM. alpha-gal syndrome vs chronic urticaria. JAMA Dermatol (2019) 155(1):115–6. doi: 10.1001/jamadermatol.2018.3970 PMC6439572 30476954

[126] Pedersen HST Sorensen JA Madsen F Linneberg A Leth-Moller KB Vestergaard C. Prevalence, predictors, and clinical relevance of alpha-gal sensitization in patients with chronic urticaria. Clin Transl Allergy (2022) 12(10):e12199. doi: 10.1002/clt2.12199 36286530 PMC9594966

[127] Maurer M Church MK Metz M Starkhammar M Hamsten C van Hage M. Galactose-alpha-1,3-galactose allergy is not a hitherto unrecognized cause of chronic spontaneous urticaria. Int Arch Allergy Immunol (2015) 167(4):250–2. doi: 10.1159/000440653 26426899

[128] Pattanaik D Lieberman P Lieberman J Pongdee T Keene AT. The changing face of anaphylaxis in adults and adolescents. Ann Allergy Asthma Immunol (2018) 121(5):594–7. doi: 10.1016/j.anai.2018.07.017 30071303

[129] Carter MC Ruiz-Esteves KN Workman L Lieberman P Platts-Mills TAE Metcalfe DD. Identification of alpha-gal sensitivity in patients with a diagnosis of idiopathic anaphylaxis. Allergy (2018) 73(5):1131–4. doi: 10.1111/all.13366 PMC7254893 29161766

[130] Gonzalez-Quintela A Dam Laursen AS Vidal C Skaaby T Gude F Linneberg A. IgE antibodies to alpha-gal in the general adult population: relationship with tick bites, atopy, and cat ownership. Clin Exp Allergy (2014) 44(8):1061–8. doi: 10.1111/cea.12326 24750173

[131] Villalta D Pantarotto L Da Re M Conte M Sjolander S Borres MP. High prevalence of sIgE to Galactose-alpha-1,3-galactose in rural pre-Alps area: a cross-sectional study. Clin Exp Allergy (2016) 46(2):377–80. doi: 10.1111/cea.12655 26450130

[132] Levin M Apostolovic D Biedermann T Commins SP Iweala OI Platts-Mills TAE. Galactose alpha-1,3-galactose phenotypes: Lessons from various patient populations. Ann Allergy Asthma Immunol (2019) 122(6):598–602. doi: 10.1016/j.anai.2019.03.021 30922956 PMC6839685

[133] Fischer J Yazdi AS Biedermann T. Clinical spectrum of alpha-Gal syndrome: from immediate-type to delayed immediate-type reactions to mammalian innards and meat. Allergo J Int (2016) 25:55–62. doi: 10.1007/s40629-016-0099-z 27226951 PMC4861743

[134] Apostolovic D Grundstrom J Perusko M Kiewiet MBG Hamsten C Starkhammar M. Course of IgE to alpha-Gal in a Swedish population of alpha-Gal syndrome patients. Clin Transl Allergy (2021) 11(10):e12087. doi: 10.1002/clt2.12087 34938441 PMC8672165

[135] Commins SP. Diagnosis & management of alpha-gal syndrome: lessons from 2,500 patients. Expert Rev Clin Immunol (2020) 16(7):667–77. doi: 10.1080/1744666X.2020.1782745 PMC8344025 32571129

[136] Kim MS Straesser MD Keshavarz B Workman L McGowan EC Platts-Mills TAE. IgE to galactose-alpha-1,3-galactose wanes over time in patients who avoid tick bites. J Allergy Clin Immunol Pract (2020) 8(1):364–7 e2. doi: 10.1016/j.jaip.2019.08.045 31520841 PMC6980488

[137] Mitchell CL Lin FC Vaughn M Apperson CS Meshnick SR Commins SP. Association between lone star tick bites and increased alpha-gal sensitization: evidence from a prospective cohort of outdoor workers. Parasit Vectors (2020) 13(1):470. doi: 10.1186/s13071-020-04343-4 32928302 PMC7490856

[138] Wilson JM Nguyen AT Schuyler AJ Commins SP Taylor AM Platts-Mills TAE. IgE to the mammalian oligosaccharide galactose-alpha-1,3-galactose is associated with increased atheroma volume and plaques with unstable characteristics-brief report. Arterioscler Thromb Vasc Biol (2018) 38(7):1665–9. doi: 10.1161/ATVBAHA.118.311222 PMC6039405 29903734

[139] Vernon ST Kott KA Hansen T Finemore M Baumgart KW Bhindi R. Immunoglobulin E sensitization to mammalian oligosaccharide galactose-alpha-1,3 (alpha-gal) is associated with noncalcified plaque, obstructive coronary artery disease, and ST-segment-elevated myocardial infarction. Arterioscler Thromb Vasc Biol (2022) 42(3):352–61. doi: 10.1161/ATVBAHA.121.316878 35045730

[140] Wilson JM McNamara CA Platts-Mills TAE. IgE, alpha-Gal and atherosclerosis. Aging (Albany NY) (2019) 11(7):1900–2. doi: 10.18632/aging.101894 PMC6503887 30958794

[141] Nunen Sv O’Connor KS Fernando SL Clarke LR Boyle R. The association between ixodes holocyclus tick bite reactions and red meat allergy. Inter Med J (2007) 37(Suppl 5):A132. doi: 10.5694/j.1326-5377.2009.tb02533.x 19413526

[142] Nunez R Carballada F Gonzalez-Quintela A Gomez-Rial J Boquete M Vidal C. Delayed mammalian meat-induced anaphylaxis due to galactose-alpha-1,3-galactose in 5 European patients. J Allergy Clin Immunol (2011) 128(5):1122–4 e1. doi: 10.1016/j.jaci.2011.07.020 21835442

[143] Jacquenet S Moneret-Vautrin DA Bihain BE. Mammalian meat–induced anaphylaxis: Clinical relevance of anti–galactose-a-1,3-galactose IgE confirmed by means of skin tests to cetuximab. J Allergy Clin Immunol (2009) 124(3):603–5. doi: 10.1016/j.jaci.2009.06.014 19733301

[144] Sekiya K Fukutomi Y Nakazawa T Taniguchi M Akiyama K. Delayed anaphylactic reaction to mammalian meat. J Investig Allergol Clin Immunol (2012) 22(6):446–7. 23101194

[145] Richards N Keshavarz B Workman L Patel J Platts-Mills TA Wilson J. Prevalence of a-gal igE and mammalian meat allergy in a COVID-19 vaccine employee cohort. J Allergy Clin Immunol (2022) 149(2):AB207. doi: 10.1016/j.jaci.2021.12.680

[146] Tjernberg I Hamsten C Apostolovic D van Hage M. IgE reactivity to alpha-Gal in relation to Lyme borreliosis. PloS One (2017) 12(9):e0185723. doi: 10.1371/journal.pone.0185723 28953957 PMC5617217

[147] Fischer J Lupberger E Hebsaker J Blumenstock G Aichinger E Yazdi AS. Prevalence of type I sensitization to alpha-gal in forest service employees and hunters. Allergy (2017) 72(10):1540–7. doi: 10.1111/all.13156 28273338

[148] Venturini M Lobera T Sebastian A Portillo A Oteo JA. IgE to alpha-gal in foresters and forest workers from la rioja, North of Spain. J Investig Allergol Clin Immunol (2018) 28(2):106–12. doi: 10.18176/jiaci.0218 29235434

[149] Westman M Asarnoj A Ballardini N Andersson N Kiewiet MBG Borres MP. Alpha-gal sensitization among young adults is associated with male sex and polysensitization. J Allergy Clin Immunol Pract (2022) 10(1):333–5 e2. doi: 10.1016/j.jaip.2021.10.018 34687938

[150] Ailsworth SM Susi A Workman LJ Ji YS Patel J Nelson MR. Alpha-gal igE prevalence patterns in the United States: an investigation of 3000 military recruits. J Allergy Clin Immunol Pract (2023) 12(1):175–184.e5. doi: 10.1016/j.jaip.2023.10.046 37918651

[151] Sagurova I Ludwig A Ogden NH Pelcat Y Dueymes G Gachon P. Predicted Northward Expansion of the Geographic Range of the Tick Vector *Amblyomma americanum* in North America under Future Climate Conditions. Environ Health Perspect (2019) 127(10):107014. doi: 10.1289/EHP5668 31670575 PMC6867274

[152] Lindgren E Gustafson R. Tick-borne encephalitis in Sweden and climate change. Lancet (9275) 2001:16–8:358. doi: 10.1016/S0140-6736(00)05250-8 11454371

[153] Hofhuis A Harms M van den Wijngaard C Sprong H van Pelt W. Continuing increase of tick bites and Lyme disease between 1994 and 2009. Ticks Tick Borne Dis (2015) 6(1):69–74. doi: 10.1016/j.ttbdis.2014.09.006 25448421

[154] Lindgren E Talleklint L Polfeldt T. Impact of climatic change on the northern latitude limit and population density of the disease-transmitting European tick Ixodes ricinus. Environ Health Perspect (2000) 108(2):119–23. doi: 10.1289/ehp.00108119 PMC1637900 10656851

[155] Platts-Mills TA Schuyler AJ Tripathi A Commins SP. Anaphylaxis to the carbohydrate side chain alpha-gal. Immunol Allergy Clin North Am (2015) 35(2):247–60. doi: 10.1016/j.iac.2015.01.009 PMC4617526 25841549

[156] Houchens N Hartley S Commins SP Claar D Saint S. Hunting for a diagnosis. N Engl J Med (2021) 384(5):462–7. doi: 10.1056/NEJMcps2017588 PMC9306225 33534979

[157] Flaherty MG Kaplan SJ Jerath MR. Diagnosis of life-threatening alpha-gal food allergy appears to be patient driven. J Prim Care Community Health (2017) 8(4):345–8. doi: 10.1177/2150131917705714 PMC5932728 28447914

[158] Carpenter A Drexler AN McCormick WD Thompson JM Kersh GJ Commins SP. Health care provider knowledge regarding alpha-gal syndrome — United States, March–May 2022. MMWR Morb Mortal Wkly Rep (2023) 72(30):809–14. doi: 10.15585/mmwr.mm7230a1 PMC10390085 37498792

[159] Brestoff JR Zaydman MA Scott MG Gronowski AM. Diagnosis of red meat allergy with antigen-specific IgE tests in serum. J Allergy Clin Immunol (2017) 140(2):608–10 e5. doi: 10.1016/j.jaci.2017.01.032 28279684

[160] Fischer J Hebsaker J Caponetto P Platts-Mills TA Biedermann T. Galactose-alpha-1,3-galactose sensitization is a prerequisite for pork-kidney allergy and cofactor-related mammalian meat anaphylaxis. J Allergy Clin Immunol (2014) 134(3):755–9 e1. doi: 10.1016/j.jaci.2014.05.051 25171869

[161] Apostolovic D Grundstrom J Nopp A Perusko M Bigdeli N Hamsten C. Do basophil activation and alpha-gal specific antibodies reflect clinical phenotypes of red meat allergic patients? Allergy (2020) 75(S109):300. doi: 10.1111/all.14507

[162] Wilson JM Platts-Mills TAE. Meat allergy and allergens. Mol Immunol (2018) 100:107–12. doi: 10.1016/j.molimm.2018.03.018 PMC6716155 29685461

[163] Patel C Iweala OI. 'Doc, will I ever eat steak again?': diagnosis and management of alpha-gal syndrome. Curr Opin Pediatr (2020) 32(6):816–24. doi: 10.1097/MOP.0000000000000955 PMC7737235 33009122

[164] Swiontek K Morisset M Codreanu-Morel F Fischer J Mehlich J Darsow U. Drugs of porcine origin-A risk for patients with alpha-gal syndrome? J Allergy Clin Immunol Pract (2019) 7(5):1687–90 e3. doi: 10.1016/j.jaip.2018.12.005 30557715

[165] Stone CA Jr. Choudhary S Patterson MF Rukasin CRF Coleman DT Phillips EJ. Tolerance of porcine pancreatic enzymes despite positive skin testing in alpha-gal allergy. J Allergy Clin Immunol Pract (2020) 8(5):1728–32 e1. doi: 10.1016/j.jaip.2019.12.004 31846796 PMC7217730

[166] Nwamara U Kaplan MC Mason N Ingemi AI. A retrospective evaluation of heparin product reactions in patients with alpha-gal allergies. Ticks Tick Borne Dis (2022) 13(1):101869. doi: 10.1016/j.ttbdis.2021.101869 34798527

[167] Hawkins RB Wilson JM Mehaffey JH Platts-Mills TAE Ailawadi G. Safety of intravenous heparin for cardiac surgery in patients with alpha-gal syndrome. Ann Thorac Surg (2021) 111(6):1991–7. doi: 10.1016/j.athoracsur.2020.07.050 PMC8019687 33031779

[168] Kuravi KV Sorrells LT Nellis JR Rahman F Walters AH Matheny RG. Allergic response to medical products in patients with alpha-gal syndrome. J Thorac Cardiovasc Surg (2022) 164(6):e411–e24. doi: 10.1016/j.jtcvs.2021.03.100 PMC9673037 33933257

[169] Mozzicato SM Tripathi A Posthumus JB Platts-Mills TAE Commins SP. Porcine or bovine valve replacement in 3 patients with IgE antibodies to the mammalian oligosaccharide galactose-alpha-1,3-galactose. J Allergy Clin Immunol Pract (2014) 2(5):637–8. doi: 10.1016/j.jaip.2014.04.016 PMC4163007 25213067

[170] Hawkins RB Frischtak HL Kron IL Ghanta RK. Premature bioprosthetic aortic valve degeneration associated with allergy to galactose-alpha-1,3-galactose. J Card Surg (2016) 31(7):446–8. doi: 10.1111/jocs.12764 PMC5013262 27238083

[171] Nourian MM Stone CA Jr. Siegrist KK Riess ML. Perioperative implications of patients with alpha gal allergies. J Clin Anesth (2023) 86:111056. doi: 10.1016/j.jclinane.2023.111056 36682226 PMC11087933

[172] Iweala OI. α-gal syndrome: busting paradigms in food allergy. Ann Internal Medicine: Clin cases (2023) 2(6):e230578. doi: 10.7326/aimcc.2023.0578 PMC11567157 39555229

[173] Unal D Eyice-Karabacak D Kutlu A Demir S Tuzer C Arslan AF. Oral immunotherapy in alpha-gal red meat allergy: Could specific IgE be a potential biomarker in monitoring management? Allergy (2023) 78(12):3241–51. doi: 10.1111/all.15840 37545316

